# Ciliary photoreceptors in the cerebral eyes of a protostome larva

**DOI:** 10.1186/2041-9139-2-6

**Published:** 2011-03-01

**Authors:** Yale J Passamaneck, Nina Furchheim, Andreas Hejnol, Mark Q Martindale, Carsten Lüter

**Affiliations:** 1 Kewalo Marine Laboratory, Pacific Biosciences Research Center, University of Hawaii, 41 Ahui Street, Honolulu, HI 96813, USA; 2 Museum für Naturkunde, Leibniz-Institut für Evolutions- und Biodiversitätsforschung an der Humboldt-Universität zu Berlin, Invalidenstraße 43, 10115 Berlin, Germany; 3 Sars International Centre for Marine Molecular Biology, University of Bergen, Thormøhlensgate 55, 5008 Bergen, Norway

## Abstract

**Background:**

Eyes in bilaterian metazoans have been described as being composed of either ciliary or rhabdomeric photoreceptors. Phylogenetic distribution, as well as distinct morphologies and characteristic deployment of different photopigments (ciliary vs. rhabdomeric opsins) and transduction pathways argue for the co-existence of both of these two photoreceptor types in the last common bilaterian ancestor. Both receptor types exist throughout the Bilateria, but only vertebrates are thought to use ciliary photoreceptors for directional light detection in cerebral eyes, while all other invertebrate bilaterians studied utilize rhabdomeric photoreceptors for this purpose. In protostomes, ciliary photoreceptors that express *c-opsin *have been described only from a non-visual deep-brain photoreceptor. Their homology with vertebrate rods and cones of the human eye has been hypothesized to represent a unique functional transition from non-visual to visual roles in the vertebrate lineage.

**Results:**

To test the hypothesis that protostome cerebral eyes employ exclusively rhabdomeric photoreceptors, we investigated the ultrastructure of the larval eyes in the brachiopod *Terebratalia transversa*. We show that these pigment-cup eyes consist of a lens cell and a shading pigment cell, both of which are putative photoreceptors, deploying a modified, enlarged cilium for light perception, and have axonal connections to the larval brain. Our investigation of the gene expression patterns of *c-opsin*, *Pax6 *and *otx *in these eyes confirms that the larval eye spots of brachiopods are cerebral eyes that deploy ciliary type photoreceptors for directional light detection. Interestingly, *c-opsin *is also expressed during early embryogenesis in all potential apical neural cells, becoming restricted to the anterior neuroectoderm, before expression is initiated in the photoreceptor cells of the eyes. Coincident with the expression of *c*-*opsin *in the presumptive neuroectoderm, we found that middle gastrula stage embryos display a positive photoresponse behavior, in the absence of a discrete shading pigment or axonal connections between cells.

**Conclusions:**

Our results indicate that the dichotomy in the deployment of ciliary and rhabdomeric photoreceptors for directional light detection is not as clear-cut as previously thought. Analyses of brachiopod larval eyes demonstrate that the utilization of *c-opsin *expressing ciliary photoreceptors in cerebral eyes is not limited to vertebrates. The presence of ciliary photoreceptor-based eyes in protostomes suggests that the transition between non-visual and visual functions of photoreceptors has been more evolutionarily labile than previously recognized, and that co-option of ciliary and rhabdomeric photoreceptor cell types for directional light detection has occurred multiple times during animal evolution. In addition, positive photoresponse behavior in gastrula stage embryos suggests that a discrete shading pigment is not requisite for directional photoreception in metazoans. Scanning photoreception of light intensities mediating cell-autonomous changes of ciliary movement may represent an ancient mechanism for regulating locomotory behavior, and is likely to have existed prior to the evolution of eye-mediated directional light detection employing axonal connections to effector cells and a discreet shading pigment.

## Background

Bilaterian photoreceptor cells are generally classified as having either ciliary or rhabdomeric morphologies, depending upon the origin of the elaborated membranes that compose their light-sensitive structures [1,2]. Recent phylogenetic and expression analyses of opsin photopigments suggest that two non-homologous phototransduction cascades characterize ciliary and rhabdomeric photoreceptors in bilaterians [3-7]. Opsins code for G-coupled protein receptors, which are localized to the elaborated membranes of photoreceptors and participate in the phototransduction pathway. Phylogenetic analyses have demonstrated that all bilaterian opsins have a monophyletic origin, with several classes, including ciliary opsins (c-opsins) and rhabdomeric opsins (r-opsins), having diverged prior to the last common ancestor of bilaterians [3,7]. In both deuterostomes and protostomes, *ciliary opsin *(*c*-*opsin*) genes have been found to be expressed in photoreceptors with a ciliary morphology, while *rhabdomeric opsin *(*r*-*opsin*) genes are expressed in photoreceptors with rhabdomeric morphology. This dichotomy suggests that these two distinct types of photoreceptors, and their associated phototransduction pathways, coexisted in the bilaterian ancestor [5-7].

Larval eyespots of protostomes are of particular interest in the study of photoreceptor evolution, since their structure has been proposed to resemble the bilaterian prototype two-celled eye [3,8]. Cerebral eyes have been defined as pigmented photoreceptor organs that (a) are positioned in the anterior region of the body in a region of *Otx *expression, (b) are connected to the anterior axonal scaffold and, (c) express *Pax6 *[3]. Based upon these criteria the eyes of many protostome larvae and of deuterostome tornaria larvae have been identified as potentially homologous structures. The discovery that *r-opsin *orthologs are expressed in larval rhabdomeric photoreceptors of the polychaete *Platynereis dumerilii *and in vertebrate retinal ganglion cells (with non-visual function), together with the development of these photoreceptors from *ath*-positive precursor cells in both animal groups, has led to the assumption of homology of these cell types [4]. In contrast to this, ciliary photoreceptors in protostomes seem to be primarily restricted to non-visual functions (for example, deep-brain photoreceptors in *P. dumerilii*), but their photopigment represents the invertebrate ortholog of the *c-opsin *expressed in visual rods and cones of the vertebrate eye, suggesting common ancestry of these ciliary photoreceptors [6]. Those protostome visual photoreceptors that do have a ciliary morphology (for example, the mantle eyes of scallops) have generally been inferred to be non-homologous evolutionary novelties [3,9,10]. Following this scenario, protostome larval cerebral eyes are predicted to deploy exclusively rhabdomeric photoreceptors expressing the *r-opsin *photopigment, while the deployment of ciliary photoreceptors in the vertebrate eye is the result of their having been substituted for rhabdomeric photoreceptors early in the evolution of the chordate lineage [3].

Brachiopods represent an intriguing group for understanding the evolution of photoreceptors, given their representation in the early fossil record of metazoans. Recent phylogenomic analyses have evidenced that brachiopods are derived members of the clade Spiralia (Lophotrochozoa), closely related to nemerteans and annelids [11,12]. The larvae of several articulate brachiopods have been described as having larval eye spots; however, the morphology of these structures has not been examined in detail [13,14].

To ascertain the nature of larval eyespots in articulate brachiopods, we have conducted a detailed morphological study of these structures in *Terebratalia transversa*. We have also assessed the presence of molecular components of eye formation. Finally, we have tested for photoresponsive behavior in early stage embryos to evaluate the possible function of an unexpected early domain of opsin gene expression.

## Results

### Ultrastructure of *Terebratalia *eyes

**Figure 1:**
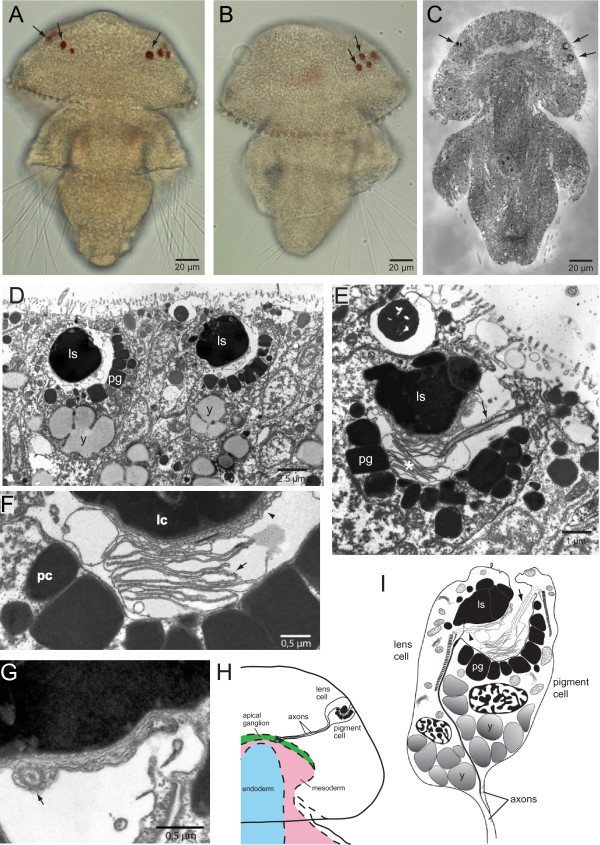
**Ultrastructure of *Terebratalia transversa *larval eyes**. **(A, B) **Brightfield microscopy of a *Terebratalia transversa *larva, with red eye spots visible in the apical lobe (black arrows). **(A)** Dorsal view. **(B)** Lateral view. **(C-F) **Ultrastructure of eyes in the larva of *Terebratalia*. **(C)** Longitudinal section through whole larva with eyes (black arrows) on either side of the apical lobe. **(D)** Two neighboring eyes with lenses (ls) in the lens cells and pigment granules (pg) in the shading pigment cells, separated by two epidermal cells. Yolk granules are present in both cells (y). **(E)** Detail of a pigment cell showing the enlarged membrane (asterisk) of its sensory cilium (arrow). **(F)** Detail of the enlarged ciliary membranes of both the lens cell (lc; black arrow) and the pigment cell (pc; black arrowhead) that fill the optical cavity. **(G) **Receptive cilia of both photoreceptor cells have a typical 9 × 2 + 2 microtubule pattern, exemplified by a cross section of the lens cell cilium (black arrow). **(H) **Reconstruction of larval eye axon tracts from serial sections. Axons from the lens cell and pigment cell extend to the apical ganglion (green), which overlays the mesoderm (pink) and endoderm (blue). **(I) **Reconstruction of a larval eye of *Terebratalia *from serial sections, consisting of a lens cell and a shading pigment cell. Notice the two enlarged sensory cilia of both cells (black arrows) and the proximal axons.

The lecithotrophic larvae of the brachiopod *Terebratalia transversa *have two rows of a variable number (three to eight) of pigmented spots, which extend in a mediolateral line slightly anterior of the dorsal rim of the apical lobe. These pigment spots, which have previously been described as eye spots [15], are visible only in the fully developed swimming larvae (Figure 1A, B; 96 hours post fertilization at 8°C). To determine whether these pigment spots are associated with photoreceptors, we performed transmission electron microscopy (TEM) on ultrathin sections of the larva (Figure 1C-G). Ultrastructural analysis demonstrated that the pigment spots are part of a simple eye composed of two putative photoreceptor cells (Figure 1D, E). One cell contains an apical intracellular lens-like structure, while the other cell contains the pigment granules (Figure 1D, E). These pigment granules are arranged in cuboidal vesicles to form a shading structure adjacent to the basal surface of the lens. Both cells possess enlarged ciliary membranes, characteristic of ciliary photoreceptor cells, located between the lens and the shading pigment (Figure 1E, F, I; Additional File 1A-K). The receptive cilia of both cells have a typical 9 × 2 + 2 pattern of microtubule organization (Figure 1G; Additional file 1L). Both photoreceptor cells also contain axons extending from the basal surface of the cell (Figure 1I). Serial ultrathin sections of whole larvae demonstrated that both photoreceptor cells of each larval eyespot have axonal connections leading to the apical concentration of nerve cells (the ganglion or 'larval brain'), supporting the cerebral nature of brachiopod larval eyes (Figure 1H).

### Orthology assignment of *Terebratalia transversa ciliary opsin*

**Figure 2:**
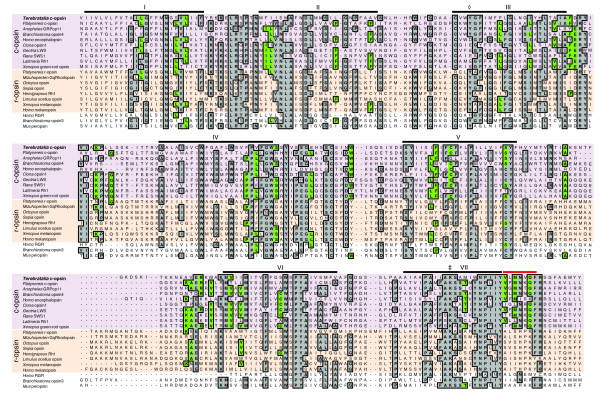
**Alignment of deduced amino acid sequences for *Terebratalia *c-opsin and representative opsins from other taxa**. Consensus amino acids (>50%) for the alignment of all opsins are shaded grey. Consensus amino acids only for the alignment of c-opsins are shaded green. In some cases the consensus residue for c-opsins differs than that for all opsins. C-opsins have a purple background, r-opsins have an orange background, and ourgroup opsins have a white background. A red bar highlights the conserved C-terminal motif of c-opsins. Transmembrane helices I-VII are marked with black horizontal lines. (‡) marks the position of the glutamate counterion and (◊) marks the conserved lysine that forms a Schiff base with retinal to form rhodopsin.

**Figure 3:**
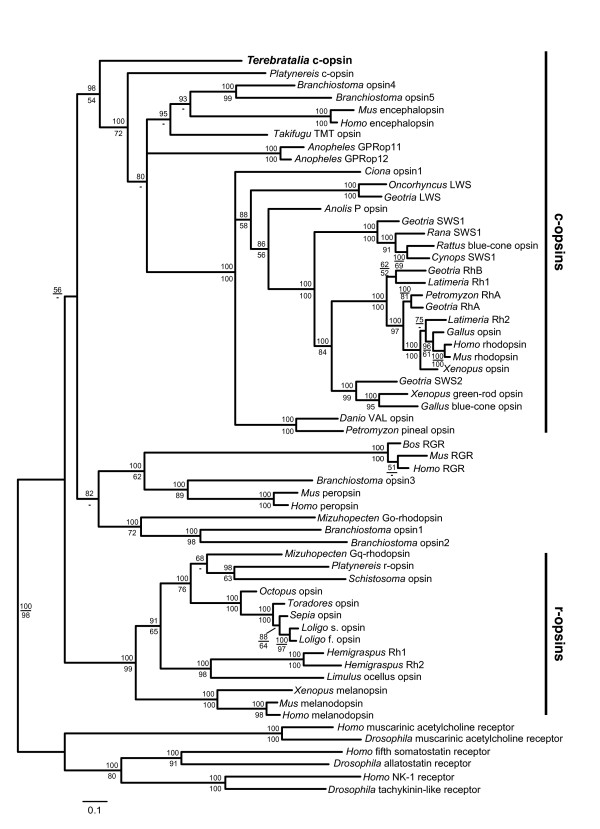
**Phylogenetic analysis of bilaterian opsins places *Terebratalia *c-opsin with c-opsins from deuterostomes and other protostomes**. Phylogram from Bayesian likelihood analysis with four independent runs of 5,000,000 generations each. Posterior probabilities are presented above branches; bootstrap support values >50% from a 1,000 replicate maximum likelihood bootstrap analysis are shown below branches.

The ciliary nature of the *Terebratalia *larval photoreceptors was further tested by examining the expression of a *ciliary opsin *(*c*-*opsin*) gene cloned from *Terebratalia*. A 422 amino acid (aa) full-length gene product was predicted from cloned cDNA sequences and aligned to protein sequences of opsin proteins from other bilaterian taxa. The predicted *Terebratalia *c-opsin protein sequence possesses seven transmembrane domains, which is characteristic of all G protein-coupled receptors, as well as a conserved lysine in transmembrane domain VII, which is specific to opsins and forms a Schiff base with retinal to form rhodopsin (Figure 2). In addition, the predicted protein possesses conserved C-terminal motifs that are unique to c-opsins (Figure 2; Additional file 2). As in *Platynereis *c-opsin, the glutamate counterion found in transmembrane domain III of many chordate c-opsins is not conserved in *Terebratalia *c-opsin (Figure 2). Phylogenetic reconstructions of the *opsin *gene family using both Bayesian and maximum likehood methods support that the gene cloned is an ortholog of other bilaterian c-opsin genes (Figure 3). We have, therefore, designated this gene *Terebratalia transversa ciliary opsin *(*Tt*-*c*-*opsin*).

### Developmental expression of *Tt-c-opsin*

**Figure 4:**
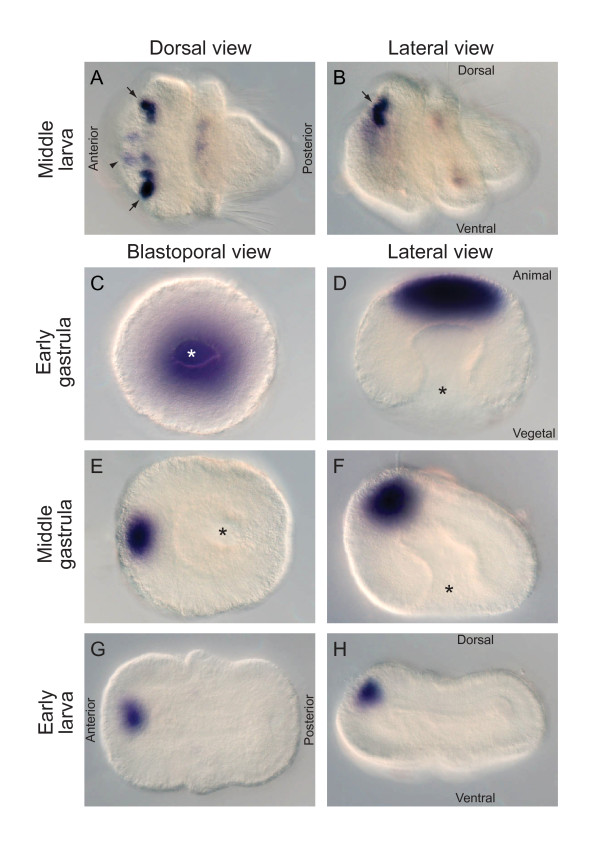
**Developmental expression of *Tt-c*-*opsin***. **(A, B) **At the middle larva stage *Tt*-*c*-*opsin *is expressed in two laterally symmetrical punctate domains, just anterior of the dorsal rim of the apical lobe (black arrows), consistent with the location of the larval eye spots, as observed in **(A)** dorsal and **(B)** lateral views. A medial-anterior domain of weak expression is also observed (black arrowhead in panel **A**). **(C, D) **At the early gastrula stage broad expression of *Tt*-*c*-*opsin *is observed at the animal pole, opposite the blastopore (*). **(E, F) **By the middle gastrula stage the blastopore (*) has shifted 90° relative to the animal pole, and *Tt*-*c*-*opsin *expression becomes localized to a medial anterior domain, which persists through the early larva stage **(G, H)**. **(C, E, G)** blastoporal views; **(D, F, H)** lateral views.

As detected by whole mount *in-situ *hybridization with a 1,584 nucleotide antisense probe, *Tt*-*c*-*opsin *expression is observed at the middle larval stage, when the three lobes (apica, mantle and pedicle) of the larva are well developed and setogenesis has commenced (Figure 4A, B). *Tt*-*c*-*opsin *is expressed in two rows of punctate domains on the dorsal surface of the apical lobe (Figure 4A, B). These expression domains are positioned anterior to the rim of the apical lobe and extend in a mediolateral line, separated at the midline. As such, the larval expression of *Tt*-*c*-*opsin *directly matches the position of the eyespots, supporting the ciliary nature of these photoreceptors.

In examining a range of embryonic stages, the first onset of *Tt*-*c*-*opsin *expression was observed, unexpectedly, at the early gastrula stage (Figure 4C, D). At this stage, *Tt*-*c*-*opsin *displays a broad domain of expression at the animal pole, the site of presumptive neurectodermal tissue, opposite the blastopore. By the middle gastrula stage the blastopore has shifted 90° relative to the animal pole, forming the anteroposterior and dorsoventral axes. At this stage *Tt*-*c*-*opsin *is localized to a subset of cells directly anterior of the apical tuft, which are derived from the animal pole (Figure 4E, F). The medial-anterior domain of expression persists through the early larval stages, when the three larval regions (apical, mantle and pedicle lobes) are first distinguishable, (Figure 4E, F). The medial-anterior domain of expression is distinct from larval expression in the presumptive eyes, and begins to fade once the trilobed larva has formed and setagenesis has commenced (Figure 4A, B).

### Developmental expression of *Tt*-*Pax6 *and *Tt*-*Otx*

**Figure 5:**
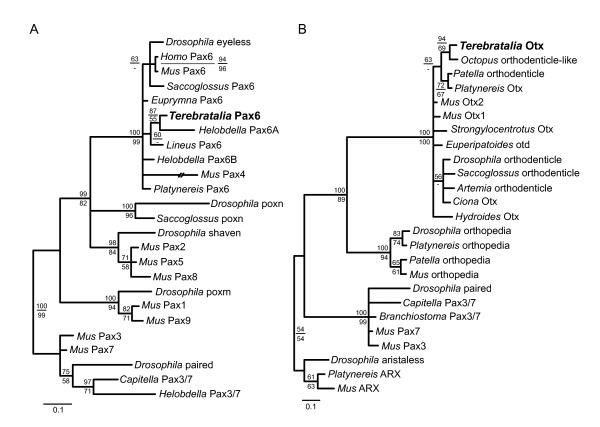
**Phylogenetic analysis of *Terebratalia *Pax6 and Otx**. **(A) **Phylogram of *Terebratalia *Pax6 and related proteins supporting orthology assignment. **(B) **Phylogram of *Terebratalia *Otx and related proteins supporting orthology assignment. Both phylograms are from from Bayesian likelihood analysis with four independent runs of 2,000,000 generations each. Posterior probabilities are presented above branches; bootstrap support values >50% from a 1,000 replicate maximum likelihood bootstrap analysis are shown below branches.

To further evaluate whether the *Terebratalia *larval photoreceptors are *bona fide *cerebral eyes we analyzed the expression of *Pax6 *and *Otx *orthologs. These two genes encode transcription factors that have been hypothesized to have conserved roles in the specification of cerebral eyes across bilaterians. For *Pax6 *a 433 aa full length gene product was predicted from cDNA sequences, and for *Otx *a 270 aa gene product was predicted from cDNA sequences. Phylogenetic reconstructions supported the orthology assignments, and we have therefore named the cloned genes *Terebratalia transversa Pax6 *(*Tt*-*Pax6*) and *Terebratalia transversa Otx *(*Tt*-*Otx*), respectively (Figure 5).

**Figure 6:**
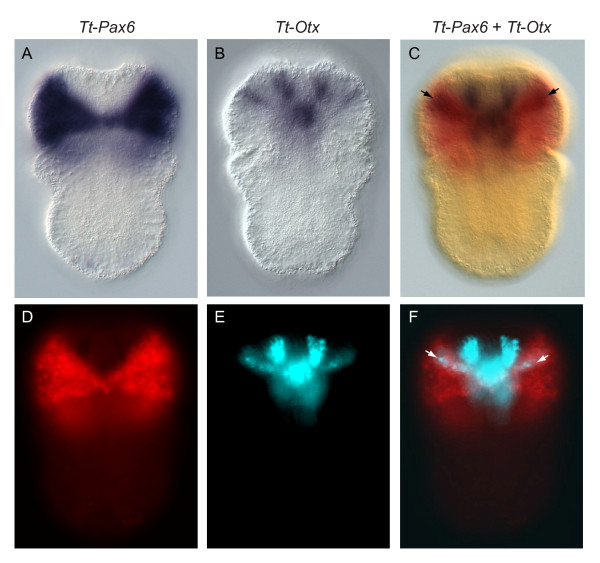
**Co-expression of *Tt-Pax6 *and *Tt*-*Otx *in the apical lobe**. **(A) **Dorsal view of *Tt*-*Pax6 *expression in the developing lobe of the early larva. **(B) **Dorsal view of *Tt*-*Otx *expression in the developing lobe of the early larva. **(C-F) **Double label *in situ *with *Tt*-*Pax6 *and *Tt*-*Otx *probes. All images are of the same embryo. **(C)** DIC image of double label *in situ *with *Tt*-*Pax6 *(red; Fast Red TR/Naphthol AS-MX) and *Tt*-*Otx *(blue; NBT/BCIP) probes, showing overlapping expression in two lateral stripes (black arrows). **(D)** Fluorescence image of *Tt*-*Pax6 *expression. **(E)** False color image of *Tt*-*Otx *expression, captured with brightfield illumination and a TRITC filter set to eliminate Fast Red TR/Naphthol AS-MX signal. **(F)** Merge of *Tt*-*Pax6 *and *Tt*-*Otx*, demonstrating overlap in two lateral stripes (white arrows).

At the early larval stage, *Tt*-*Pax6 *is expressed in two broad triangular domains in the dorsal epidermis of the presumptive apical lobe (Figure 6A, D). These two domains of expression meet in a narrow region at the midline, and expand both anteriorly and, to a lesser extent, posteriorly towards the lateral regions of the embryo.

At the early larval stage there are five domains of *Tt*-*Otx *expression on the dorsal side of the forming apical lobe (Figure 6B, E). One medial spot of expression is located near the posterior edge of the apical lobe. Two sets of laterally symmetrical stripes of expression are located in the anterior region of the apical lobe. The more anterior pair of stripes is slightly offset from the midline, and the stripes extend in an anteroposterior orientation. The second pair of stripes is located more posteriorly, and the stripes extend at approximately a 45° angle relative to the anteroposterior axis, centered on the medial spot of expression. These more posterior stripes extend to the lateral sides of the embryo and do not contact the medial spot of expression.

Double labeling of *Tt*-*Pax6 *and *Tt*-*Otx *demonstrated that the two genes are co-expressed only in a narrow region on the dorsal surface of the developing apical lobe at the early larval stage (Figure 6C, H). At this stage the posterior lateral bands of *Tt*-*Otx *expression overlap with the anterior edges of the *Tt*-*Pax6 *expression domain (Figure 6H). The region of overlap extends mediolaterally along the dorsal surface of the apical lobe, and is located approximately one-third of the length of the apical lobe posterior of the anterior end of the embryo. The placement of the overlap in *Tt*-*Pax6 *and *Tt*-*Otx *expression is, therefore, consistent with these two genes being co-expressed in the site of presumptive larval eyespot formation. The domains of overlapping *Tt*-*Pax6 *and *Tt*-*Otx *expression are in stripes of contiguous cells, rather than the punctate expression observed for *Tt*-*c*-*opsin *expression at the middle larva stage.

**Figure 7:**
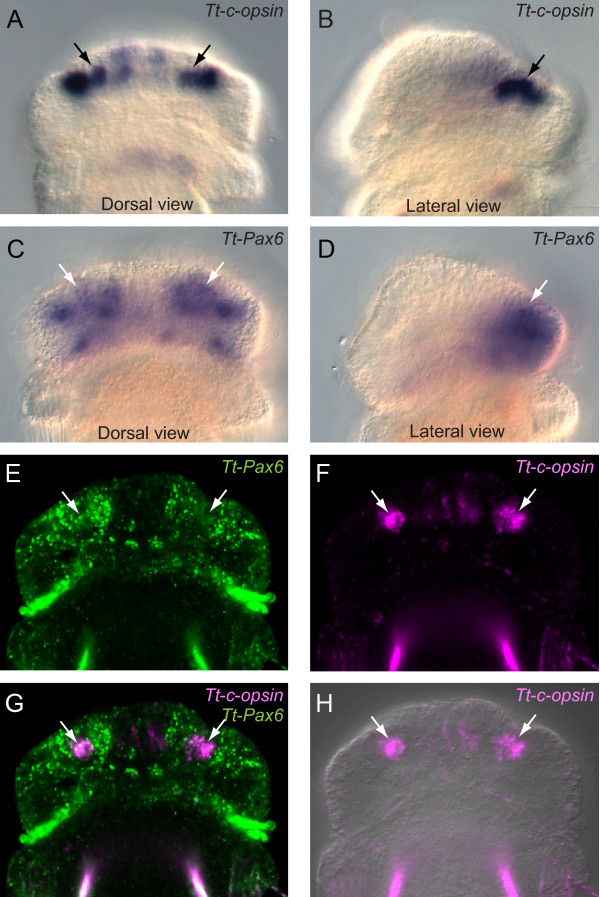
**Overlapping experession of *Tt*-*c*-*opsin *and *Tt*-*Pax6 *in the apical lobe**. **(A) **Dorsal and **(B) **lateral views of *Tt*-*c*-*opsin *expression in the apical lobe of the middle larva stage. Strong expression is observed in two lateral lines of punctate staining, matching the position of the larval eyes (black arrows). **(C, D) ***Tt*-*Pax6 *is expressed in a broader domain at the same stage. The anterior edge of the *Tt*-*Pax6 *expression domain overlaps with that of *Tt*-*c*-*opsin *(white arrows). **(E-H) **Two-color fluorescent *in situ *with *Tt*-*Pax6 *and *Tt*-*Otx *probes. All images are of the same embryo. **(E)** Single confocal section of *Tt*-*Pax6 *expression (white arrows). Fluorescence at the edge of the apical lobe (white arrowheads) is due to endogenous autofluorescence of the vesicular bodies. Fluorescence between the mantle and pedicel lobes (blue arrowheads) is due to probe trapping. **(F)** Single confocal section of *Tt*-*c*-*opsin *expression (white arrows). Fluorescence between the mantle and pedicel lobes (blue arrowheads) is due to probe trapping. **(G)** Single confocal section showing overlapping *Tt*-*Pax6 *(green) and *Tt*-*c*-*opsin *(purple) expression (white arrows). (H) Single confocal section of *Tt*-*c*-*opsin *expression (purple; white arrows) overlayed with a DIC image to show the morphological position of expression domains.

By the middle larval stage, when setae have begun to form, *Tt*-*Pax6 *expression was observed in the dorsal half of the apical lobe, extending from the posterior edge of the lobe to just anterior of the rim of the lobe (Figure 7A, B). The anterior portion of this expression pattern overlaps with the site of *Tt*-*c-opsin *expression (Figure 7C, D).

### Embryonic photoresponse behavior

Expression of opsin genes in tissues other than pigmented eyes (for example, *c*-*opsin *expression in the *Platynereis *adult brain [6]) has generally been viewed as related to detection of non-directional light signals, such as diurnal cycles, due to the lack of a shading pigment to block off-axis illumination of the photoreceptor [6,16]. *Terebratalia *embryos hatch as ciliated blastulae, and by the middle gastrula stage, when anteroposterior polarity is first established, their swimming is spiral with a left-handed rotation about the anteroposterior axis. To evaluate whether early *Tt*-*c*-*opsin *might be part of an early directional photoreceptor, we tested for photoresponsive behavior in middle gastrula stage embryos, when *Tt*-*c*-*opsin *expression becomes restricted to the medial anterior domain.

**Figure 8:**
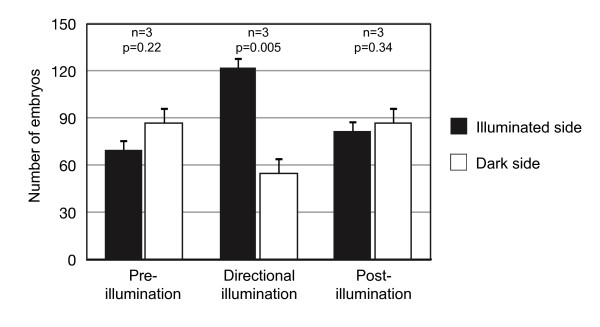
**Bar graph of results from photoresponse behavior experiments with middle gastrula stage embryos**. Distributions of embryos on illuminated and dark halves of the phototaxis chamber are shown for sampling periods before, during, and after the period of directional illumination. Middle gastrula embryos preferentially moved towards the light when exposed to directional illumination.

Middle gastrula stage embryos were placed in a phototaxis chamber where directional illumination could be introduced from one side of the chamber. Prior to the initiation of directional illumination the embryos showed no significant difference in distribution between the two sides of the chamber (Figure 8; Additional file 3; *P *= 0.22; n = 3). 20 to 25 minutes after initiation of directional illumination, embryos showed a significant bias in distribution towards the illuminated side of the chamber (Figure 8; Additional file 4; *P *= 0.005; n = 3). 5 to 10 minutes after extinguishment of directional illumination the embryos had returned to an equal distribution between the two sides of the chamber (Figure 8; Additional file 5; *P *= 0.34; n = 3). Distributions before and after illumination were equivalent (*P *= 0.17), while both differed significantly from the distribution during illumination (*P *= 0.02 and *P *= 0.01, respectively). Although the behavior of three-lobed stage larvae with eyespots in culture suggests that they are also photoresponsive, measurements in a phototaxis chamber did not yield a quantifiable response to directional illumination (data not shown).

## Discussion

The diversity of eyes throughout the bilaterians has led to an ongoing debate regarding their evolution. Based upon the observation that photoreceptive structures generally have either ciliary or rhabdomeric morphology, Eakin [1,9] proposed two lines of photoreceptor evolution, with the rhabdomeric type having evolved from the ciliary type early in the protostome lineage. Vanfleteren and Coomans [17] concluded that rhabdomeric photoreceptors were a subset of ciliary photoreceptors, and thus photoreceptors are homologous across bilaterians. In contrast to both these interpretations, Salvini-Plawen and Mayr [10,18] interpreted the diversity of photoreceptors as evidence of polyphyletic origins, postulating that photoreceptors have evolved many times independently in bilaterians.

The advent of molecular genetics led to the unexpected discovery that homologs of several transcription factor families are required for eye formation in both vertebrates and *Drosophila*, including otx/orthodenticle, Six3/sine oculis, and Pax6/eyeless. The role of *Drosophila *eyeless and vertebrate Pax6 genes in eye development, as well as the apparent functional equivalence of numerous orthologs from diverse bilaterian taxa, prompted Gehring and Ikeo [8] to propose a monophyletic origin for bilaterian eyes. While the homology of eyes based upon a role for Pax6 as a "master control gene" has been criticized as an oversimplification [19] it appears that Pax6 is a key player in eye development of most bilaterians.

The potential for the phylogenetic distribution of photoreceptors with ciliary and rhabdomeric morphologies to be of evolutionary significance has been bolstered by the recognition that the two types of photoreceptors are characterized by the expression of distinct classes of opsin genes. In all cases examined to date, photoreceptors with ciliary morphology have been shown to express *c*-*opsin *class genes and rhabdomeric photoreceptors express *r*-*opsin *genes (with the exception of the scallop *Mizuhopecten*, where the mantle eyes have ciliary morphology, but have been shown to express a Go opsin [20]).

Based upon the occurrence of rhabdomeric photoreceptors in the larval eyes of annelids, arthropods and hemichordates, Arendt and Wittbrodt [3] have suggested that while rhabdomeric and ciliary photoreceptors co-existed in the last common ancestor of bilaterians, the use of rhabdomeric photoreceptors is the ancestral condition, with ciliary photoreceptors having been co-opted for vision within the vertebrate lineage. To date, the expression of *c*-*opsin *class genes has been investigated in only two protostomes, the annelid *Platynereis *[6] and the honeybee *Apis *[16]. In both cases, expression was observed in the brain, and due to the lack of pigmentation in both these structures they have been inferred to be non-visual photoreceptors. While we observed early expression of *Tt*-*c*-*opsin *in the presumptive neuroectoderm at gastrula and early larval stages of *Terebratalia *development, we did not observe expression in the apical ganglion, which may be comparable to the brain of other protostomes.

### Ciliary larval eyes in *Terebratalia*: novelty, substitution, or ancestral condition?

By ultrastructural analysis we have demonstrated that the larval eyespots of the brachiopod *Terebratalia *are composed of two photoreceptors cells, one forming the lens cell and the other the pigmented shading cell. Both cells possess elaborated ciliary membranes in the intercellular space between the lens and the pigment granules, as well as axonal projections extending to the apical ganglion. Supporting the ciliary nature of the larval photoreceptors, we observed that a *c*-*opsin *gene is expressed specifically at the position of the eyespots in the larva. Together these results evidence that the *Terebratalia *larvae possess cerebral eyes that are capable of directional light detection, and that are of a ciliary nature, based upon both morphological and molecular criteria.

Given that Brachiopoda group within the protostome clade Spiralia (Lophotrochozoa) in molecular phylogenies [11,12,21], our results provoke the question of whether ciliary photoreceptors play a more important role in protostome eye evolution than previously thought. Whereas polychaete trochophore larvae deploy rhabdomeric photoreceptors (*r-opsin *expression) directly connected to locomotory cilia for directional movement [22], the morphology of the *Terebratalia *eyespots strongly suggests that brachiopods use ciliary photoreceptors with *c-opsin *expression for the same purpose. We cannot rule out the possibility that an *r*-*opsin *homolog is also expressed in the larval eyes of *Terebratalia*, as our attempts to clone such a gene with degenerate primers were unsuccessful.

If manifold independent acquisition of photoreceptor cells in Bilateria can be ruled out by the duality of the existing phototransduction cascades and their respective photoreceptor cell types (rPRCs *versus *cPRCs) based on bilaterian opsin phylogeny [3], a functional switch from visual to non-visual roles (or *vice versa*) for the two commonly inherited photoreceptor cell types may have happened several times independently in bilaterian evolution. Such a scenario has already been proposed for the evolution of the visual rods and cones in the vertebrate retina as derivatives of non-visual ciliary deep-brain photoreceptors of invertebrates, such as those in the annelid *Platynereis *[6].

For the evolution of brachiopod larval eyes this suggests that brachiopods have retained ciliary photoreceptor cells from the bilaterian ancestor, and deployed these for directional light detection in their larval eye spots. Given that ciliary photoreceptor cells are a plesiomorphic trait, rather than being independently evolved in brachiopods, three alternative scenarios may account for the ciliary nature of the larval eyes in *Terebratalia*: I) The larval eyes of *Terebratalia *are evolutionary novelties, unrelated to the rhabdomeric cerebral eyes of other larvae in the clade Spiralia; II) The cerebral eyes of *Terebratalia *are homologous to the larval eyes of other members of the clade Spiralia (for example, polychaetes and mollusks), with ciliary photoreceptor cells having been substituted for the rhabdomeric photoreceptor cells observed in the larval eyes of other taxa; III) Larval eyes with ciliary photoreceptors are the ancestral condition for protostomes and have been inherited by *Terebratalia*.

**Figure 9:**
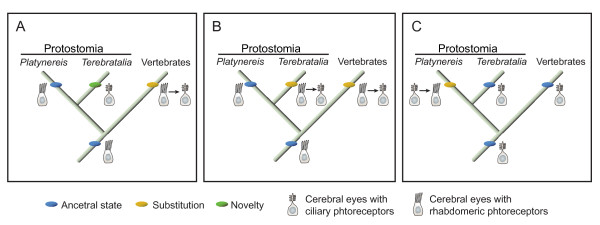
**Alternative hypothesis on the evolution of photoreceptor deployment in cerebral eyes**. Schematic representation of three hypotheses accounting for the deployment of ciliary photoreceptors in the cerebral eyes of *Terebratalia *and vertebrates, versus rhabdomeric photoreceptors in *Platynereis *and other protostomes. **(A) **Deployment of rhabdomeric photoreceptors as the ancestral state in cerebral eyes, with the larval eyes of *Terebratalia*, containing ciliary photoreceptors, representing an evolutionary novelty. The deployment of ciliary photoreceptors is the result of a substitution (with ciliary photoreceptors having replaced rhabdomeric photoreceptors in the cerebral eyes) early in the chordate lineage. **(B) **Larval eyes in *Terebratalia *are homologous to the cerebral eyes in other protostomes, but ciliary photoreceptors have been substituted for rhabdomeric photoreceptors, as in the vertebrates. **(C)** Ciliary photoreceptors in cerebral eyes represent the ancestral condition, inherited by *Terebratalia *and vertebrates. Deployment of rhabdomeric photoreceptors in the cerebral eyes of *Platynereis *and other protostomes are the result of substitution events.

Visual photoreceptors with ciliary-type morphologies have been identified in several protostomes; however, these organs have generally been regarded as evolutionary novelties due to their morphological locations (for example,the branchial crown eyes in polycheates [23,24], and the mantle eyes of scallops [25]. The ciliary larval eyes of *Terebratalia *could likewise represent an evolutionary novelty that has recruited a ciliary photoreceptor to form a cerebral larval eye comparable to, but not homologous with, the rhabdomeric larval eyes of other spiralians (Figure 9A).

Although the ciliary photoreceptor cells in the larval eyes of *Terebratalia *seem not to be homologous to the rhabdomeric photoreceptor cells in the larval eyes of *Platynereis *and other protostomes, the possibility exists that there is homology at the level of the larval eye. If the regulation of eye specification is distinct from that of photoreceptor cell differentiation, then the ciliary photoreceptor cell may have been substituted for the rhabdomeric photoreceptor cell in a homologous larval eye (Figure 9B). In a variety of protostomes and deuterostomes, *Pax6 *and *Otx *(among other transcription factor genes) have been shown to be involved in eye specification and differentiation, or to be expressed in cerebral eyes or their precursors (for example, *Drosophila *[26,27], *Platynereis *[4,28], mouse [29,30]). Expression of *Pax6 *and *Otx *in the precursors of the *Terebratalia *larval photoreceptors suggests that the genetic network underlying the formation of the larval eyes in *Terebratalia *shares common features with the network underlying the specification of rhabdomeric larval eyes in other protostomes. In *Terebratalia*, ciliary photoreceptor cells, and *c*-*opsin *expression, may have been co-opted to supplant the rhabdomeric photoreceptor cells in homologous ancestral eyes. By analogy, ontogenetic changes from one photoreceptor cell type to the other have been observed in the larval cerebral eyes of the gastropod mollusk *Aporrhais pespelecani*, in which the photoreceptor cell initially has a ciliary morphology, only to later develop microvilli, taking on a mixed-type morphology [31]. Although the expression of opsin genes in the *Aporrhais *eye is unknown, the ontogentic alterations it undergoes suggest that photoreceptor cell morphology may be decoupled from cerebral eye specification.

Finally, it should be considered that eyes with ciliary photoreceptors represent the ancestral state for Spiralia, and possibly for Bilateria (Figure 9C). Arendt and Wittbrodt [3] proposed that cerebral larval eyes with rhabdomeric photoreceptors represent the ancestral state for Bilateria, based in part upon the occurrence of eyes with this morphology in annelids, mollusks, platyhelminthes, crustaceans and hemichordates. As stated above, eyes with ciliary photoreceptors in protostomes have been regarded as evolutionary novelties or "phylogenetically young organs" [10]. However, it should be noted that cerebral larval eyes with ciliary morphology have been described from gastropod mollusks [32-35], and cerebral larval eyes with both ciliary and rhabdomeric photoreceptor cells have been described in both platyhelminthes [36,37], and the hemichordate *Ptychodera flava *[38]. Likewise, photoreceptors with ciliary morphology, which may be cerebral eyes, have been described from the larvae of ectoprocts [39,40] and an entoproct [41], both of which are members of the protostome clade Spiralia, along with brachiopods, annelids, mollusks, and platyhelminthes [12].

While ciliary photoreceptors are not the predominant form in the larval cerebral eyes of protostomes, they are found in a phylogenetically diverse range of taxa. It should, therefore, be considered that the use of ciliary photoreceptors in eyes may be an ancestral condition for Spiralia, and possibly Bilateria. In contrast to this hypothesis, Arendt *et al*. [6] proposed that localization of unpigmented ciliary receptors to the deep-brain, as seen in *Platynereis*, represents the ancestral state for bilaterians. However, Nilsson [42] has recently suggested that such photoreceptors might historically been associated with shading pigments for use in directional photoperception (that is, pigmented eyes). If this is the case, then the ciliary eyes of *Terebratalia *and other spiralians may represent an ancestral condition, rather than being evolutionary novelties.

### A minimal photoreceptor mediating early photoresponse behavior

The photoresponse behavior of the gastrula stage embryo is a somewhat surprising result. This photoresponse may be attributed to one of two alternative mechanisms, phototaxis or photokinesis. Phototaxis, movement to or away from light, is associated with directional photoreceptors, which are generally viewed as requiring an associated shading pigment to block off-axis light [3,43]. Nilsson [41] has proposed that scanning photoperception, wherein movement of the photoreceptor allows detection of differential light intensities, may have provided a primitive mechanism detecting the directionality of light; however, relatively few examples of such a photoreceptor have been described. Although photoreceptors without associated shading pigments have been described from a variety of metazoans, they have almost always been attributed to having non-visual roles in monitoring ambient luminance, such as for detection of diurnal or lunar cycles of illumination. We propose that in middle gastrula stage *Terebratalia *embryos, the yolk of the lecithotrophic embryo acts as a partial shading pigment to block off axis light, while the spiral swimming patterns serves to generate a scanning movement for directional sampling of illumination intensities. Alternatively, accumulation of embryos on the brightly illuminated side of the chamber may represent a photokinetic response; that is, a change in movement in response to light, independent of photoreceptor orientation. In this scenario, a slowing in the speed of ciliary beating of c-opsin expressing cells in response to increased illumination could cause the distribution of embryos to shift towards the light source. Further experiments are required to resolve whether phototaxis of photokinesis is responsible for the observed positive photoresponse behavior of the middle gastrula stage embryos.

Irrespective of the mechanism of the photoresponse, it is of particular interest that the positive photoresponse behavior of the middle gastrula stage embryos occurs before the onset of neuronal differentiation. At this stage the *c*-*opsin *expressing cells and their neighbors constitute a ciliated columnar epithelium without axonal connections. This suggests that the photoresponsive cells may also serve as direct behavior effectors, either through alteration of ciliary beating patterns or through mediation of changes in the orientation of the elongate cilia of the apical tuft to alter the direction of swimming in response to light. An analogous case is that of the parenchymella larvae of certain sponges, which show positive and/or negative phototaxis behavior in the absence of a nervous system or gap junctions for cell-to-cell communication. In sponge larvae, phototactic behavior is thought to be mediated by a posterior ring of pigmented cells [44-46]. However, the mechanism by which changes in ciliary behavior in response to variable illumination may affect larval swimming behavior is not fully understood [45,46]. In addition, no definitive opsin ortholog has yet been isolated from sponges, although over 200 rhodopsin-related GPCR genes have been identified in the recently published genome of *Amphimedon queenslandica *[47]. It has been hypothesized that sponge larvae may use a non-homologous mechanism for photoreception, such as flavin [48], carotenoid [48], or cytochrome c oxidase [49]. A minimal photoreceptor cell has also been proposed to occur in the larva of the box jellyfish *Tripedalia cystophora*, based upon morphology [50]. Pigmented cells in these larvae have rhabdomeric microvilli and a motor cilium, and occur in the absence of a nervous system. However, opsin expression has not been shown for these cells, nor has phototactic behavior been demonstrated for the larvae.

Our results suggest that in *Terebratalia *middle gastrula stage embryos, *c*-*opsin *expressing cells at the anterior of the embryo may be mediating a positive phototactic response in the absence of discrete shading pigments or axonal connections between cells. As such, the *Terebratalia *gastrula may utilize one of the simplest systems of directional photoperception and effector behavior described to date in bilaterians. Additional studies will be required to understand the details of this phototactic behavior, including the effect of changes in light intensity on rates of ciliary beating, and the potential role of *c*-*ospin *expression in mediating this behavior.

## Conclusions

Using both morphological and molecular analyses we have provided evidence that the larval cerebral eyes of the brachiopod *Terebratalia transversa *have ciliary photoreceptors expressing *c*-*opsin *as a photopigment. The co-expression of *Pax6 *and *Otx *in the domains where the larval eyes will form suggests that the larval eyes of *Terebratalia *share common patterning mechanismswith the cerebral eyes of other bilaterians, and may be homologous to the rhabdomeric larval eyes of other spiralian taxa. These results suggest that the deployment of ciliary and rhabdomeric photoreceptors for directional photodetection in the Bilateria has been more evolutionarily labile than current hypotheses of eye evolution have proposed.

The timing and location of early *Tt*-*c*-*opsin *expression is coincident with a positive photoresponse behavior in the unpigmented middle gastrula stage embryo prior to neural differentiation. We propose that c-opsin may facilitate photosensitivity of a simple scanning photoreceptor, in which phototaxis is facilitated through the autonomous activity of cells that act as both photoreceptors and ciliated behavioural effectors. This would represent a very simple system similar to hypothesized intermediates in the evolution of directional photoreception.

## Methods

### Animal culture

Gravid adult *Terebratalia transversa *were obtained at Friday Harbor Laboratories (San Juan Island, WA, USA), and *in vitro *fertilization was performed following established protocols from Reed [51]. Briefly, gametes were dissected from gravid animals and ovaries were macerated through 250 μm Nitex mesh (Sefar Inc, Depew, NY, USA to separate oocytes. Oocytes were allowed to settle in a beaker of filtered seawater, washed several times with filtered seawater, and maintained in a flow-through sea table until germinal vesicle breakdown and shedding of follicle cells were observed though a stereomicroscope (approximately six to eight hours). Testes were macerated in filtered seawater made alkaline to pH 9.8 with 1 N sodium hydroxide, and the solution was monitored on a compound microscope until sperm became visibly motile (approximately 20 minutes). A total of 5 ml of sperm solution was added to oocytes in 250 ml of filtered seawater, and washed out after one hour. Embryos were collected at different stages up to the competent larva.

### Transmission electron microscopy

Embedded three-lobed stage larvae of *Terebratalia transversa *(Sowerby, 1846) were kindly provided by Stephen A. Stricker (Department of Biology, University of New Mexico, Albuquerque, NM, USA) for further investigation. Details of the fixation process have been published elsewhere [52]. Ultrathin serial sections of about 70 nm thickness were cut with a diamond knife on a Leica Ultracut UCT microtome (Leica, Wetzlar, Germany), subsequently stained with 1% uranyl acetate (50 minutes/30°C) and lead citrate (25 minutes/25°C) in a Leica EM Stain and examined with LEO 912 Omega (Zeiss, Oberkochen, Germany) and Philips CM 120 Biotwin (FEI, Eindhoven, The Netherlands) transmission electron microscopes. Photographs were taken on Kodak EM 4489 negative films digitised with a Silver Fast Mikrotek ScanMaker 1000 × l (Lasersoft Imaging, Kiel, Germany) or on Ditabis photoplates digitised on a Ditabis scanner (DITABIS, Pforzheim, Germany). Digitized photographs were processed and arranged using Adobe CS3 (Adobe Systems Inc., San Jose, CA, USA).

### Gene isolation

Fragments of *Tt-c-opsin *and *Tt-Pax6 *were amplified by degenerate PCR using as template complementary DNA from mixed stages. Degenerate primers for semi-nested amplification of *Tt-c-opsin*: WSNTAYATHATHTTYYTITTYRTITTY, forward [6]; GCNTGGWSICCITAYGC, nested forward; NCKRAAYTGIKTRTTCATIMMIACRTADAT, reverse [6]. Degenerate primers for nested amplification of *Tt-Pax6*: GTNAAYCARYTNGGNGGNGT, forward; GTNAAYGGN MGNCCIYTICC, nested forward; RTCNCKDATYTCCCANGCRAA, reverse; TTNGGYTTNSWNCCNCCDAT, nested reverse. *Tt-Otx *was initially identified from an EST clone sequenced for phylogenomic analysis [11]. Full length cDNAs were obtained by rapid amplification of cDNA ends using the SMART RACE kit (Clontech Laboratories, Inc., Mountain View, CA, USA). The use of several degenerate primer sets targeted against *r-opsin *did not lead to the amplification of a gene fragment.

### Whole mount *in situ *hybridization

*In situ *hybridizations were carried out using an established protocol [53]. NBT/BCIP stained larvae were imaged under brightfield Nomarski optics with a Zeiss Axiocam HR mounted on a Zeiss Axioskop 2 mot plus. Double label *in situ *hybridizations were imaged with a Hamamatsu Orca mounted on a Zeiss Imager Z1. NBT/BCIP staining was imaged with brightfield illumination and a 45/Texas Red filter to minimize signal from the HNPP/FastRed precipitate. HNPP/FastRed staining was imaged by fluorescence illumination with a 45/Texas Red filter. The NBT/BCIP image was inverted and false-colored in ImageJ [54], and the NBT/BCIP and HNPP/FastRed images where merged to determine regions of co-expression.

### Whole mount fluorescent *in situ *hybridization

Two-color fluorescent *in situ *hybridizations were carried out with TSA Plus Fluorescence Kits (PerkinElmer, Waltham, MA, USA). Fixed larvae with hybridized with Digoxigenin-11-UTP (Roche Applied Science, Indianapolis, IN, USA) labelled *Tt-c-opsin *and Fluorescein-12-UTP (Roche Applied Science, Indianapolis, IN, USA) labelled *Tt*-*Pax6 *probes at 2.5 ng/μl each, for 48 hours at 60°C. Larvae were incubated overnight with Anti-digoxigenin-POD antibody (Roche Applied Science, Indianapolis, IN, USA) at a 1:1,000 dilution in 1× Blocking Reagent (Roche Applied Science, Indianapolis, IN, USA) overnight at 4°C, and stained with TSA Plus Cyanine 5 at a 1:100 dilution for 60 minutes at room temperature. Following staining, peroxidase activity was extinguished by incubation in 2.7% hydrogen peroxide in 1× PBS buffer for 90 minutes. Subsequently, larvae were incubated overnight with Anti-fluorescein-POD antibody (Roche Applied Science, Indianapolis, IN, USA) at a 1:1,000 dilution in 1× Blocking Reagent (Roche Applied Science, Indianapolis, IN, USA) overnight at 4°C, and stained with TSA Plus Tetramethylrhodamine at a 1:100 dilution for 60 minutes at room temperature. Stained larvae were washed for 48 hours with multiple exchanges of 1× PBS buffer, and cleared with 80% glycerol. Confocal imaging was performed using a Zeiss LSM 710 microscope (Carl Zeiss MicroImaging, Inc., Thornwood, NY, USA) with a 40×/1.3 NA oil immersion objective.

### Phylogenetic analysis

The deduced amino acid sequences for *Terebratalia *c-opsin, Pax6 and Otx, along with those for representative related proteins from other taxa, retrieved from NCBI (html://ncbi.nlm.nih.gov/; accession numbers listed below), were aligned with MUSCLE [55]. The resultant alignments were corrected by eye and non-conserved regions excluded from further analyses. For each dataset, the best-fit model of protein evolution was determined with ProtTest [56] and was employed in all subsequent analyses. For the c-opsin alignment, the WAG+I+G model was determined to be the best-fit, while for both Pax6 and Otx the JTT model was the best-fit. Bayesian likelihood analysis of all datasets was performed with v3.1.2 of Mr. Bayes [57,58]. For c-opsin, four independent runs of 5,000,000 generations each were performed, and a burn-in of 1,000,000 generations was applied. For Pax6 and Otx, four independent runs of 2,000,000 generations each were performed, and a burn-in of 500,000 generations was applied. For all datasets, bootstrap support values were derived from a 1,000 replicate maximum likelihood analysis performed in PhyML [59].

### Accession numbers for sequences included in phylogenetic analyses

#### opsins

*Anolis *P opsin (AAD32622.1); *Anopheles *GPRop11 (XP_312503.3); *Anopheles *GPRop12 (XP_312502.2); *Bos *RGR (NP_786969.1); *Branchiostoma *opsin1 (BAC76019.1); *Branchiostoma *opsin2 (BAC76020.1); *Branchiostoma *opsin3 (BAC76023.1); *Branchiostoma *opsin4 (BAC76021.1); *Branchiostoma *opsin5 (BAC76022.1); *Ciona *opsin1 (BAB68391.1); *Cynops *SWS1 (BAB79499); *Danio *VAL_opsin (NP_571661.1); *Drosophila *allatostatin_receptor (AAF05299.1); *Drosophila *muscarinic acetylcholine receptor (AAA28676.1); *Drosophila *Tachykinin-like receptor (NP_524304.2); *Gallus *blue-cone opsin (NP_990848); *Gallus *opsin (P22328); *Geotria *LWS (AAR14680); *Geotria *RhA (AAR14682); *Geotria *RhB (AAR14683); *Geotria *SWS1 (AAR14684); *Geotria *SWS2 (AAR14681); *Hemigrapsus *Rh1 (Q25157); *Hemigrapsus *Rh2 (Q25158); *Homo *encephalopsin (NP_055137.1); *Homo *fifth somatostatin receptor (BAA04107.1); *Homo *melanopsin (NP_150598.1); *Homo *muscarinic acetylcholine receptor (CAA68560.1); *Homo *NK-1 receptor (AAA59933.1); *Homo *peropsin (NP_006574.1); *Homo *RGR (P47804); *Homo *rhodopsin (NP_000530.1); *Latimeria *Rh1 (AAD30520.1); *Latimeria *Rh2 (AAD30519); *Limulus *ocellus opsin (P35361); *Loligo *f. opsin (P24603); *Loligo *s. opsin (Q17094); *Mizuhopecten *Go-rhodopsin (O15974.1); *Mizuhopecten *GqRhodopsin (O15973); *Mus *encephalopsin (NP_034228.1); *Mus *melanopsin (NP_038915.1); *Mus *peropsin (NP_033128.1); *Mus *RGR (NP_067315.1); *Mus *rhodopsin (NP_663358); *Octopus *opsin (P09241); *Oncorhyncus *LWS (AAP58346); *Petromyzon *RhA (Q98980); *Petromyzon *pineal opsin (O4249); *Platynereis *c-opsin (AAV63834.1); *Platynereis *r-opsin (CAC86665.1); *Rana *SWS1 (BAA96828); *Rattus *blue-cone opsin (NP_112277); *Schistosoma *opsin (AAF73286.1); *Sepia *opsin (O16005); *Takifugu *TMT opsin (AAL83430.1); *Terebratalia *c-opsin (HQ679623); *Todarodes *opsin (P31356); *Xenopus *green-rod opsin (AAO38746); *Xenopus *melanopsin (AAC41235.1); *Xenopus *opsin (P29403)

#### Pax6

*Capitella *Pax3/7 (ABC68267.1); *Drosophila *eyeless (NP_524628.2); *Drosophila *paired (NP_523556.1); *Drosophila *poxm (NP_001036687.1); *Drosophila *poxn (NP_476686.1); *Drosophila *shaven (NP_524633.3); *Euprymna *Pax6 (AAM74161.1); *Helobdella *Pax3/7 (ABI17942.1); *Helobdella *Pax6A (ABN09915.2); *Helobdella *Pax6B (ABN09916.2);

*Homo *Pax6 (NP_000271.1); *Lineus *Pax6 (CAA64847.1); *Mus *Pax1 (NP_032806.2); *Mus *Pax2 (NP_035167.3); *Mus *Pax3 (NP_032807.3); *Mus *Pax4 (NP_035168.1); *Mus *Pax5 (NP_032808.1); *Mus *Pax6 (NP_038655.1); *Mus *Pax7 (NP_035169.1); *Mus *Pax8 (NP_035170.1); *Mus *Pax9 (NP_035171.1); *Platynereis *Pax6 (CAJ40659.1); *Saccoglossus *Pax6 (NP_001158383.1); *Saccoglossus *poxn (NP_001158393.1); *Terebratalia *Pax6 (HQ679621)

#### Otx

*Artemia *orthodenticle (ACQ90718.1); *Branchiostoma *Pax3/7 (ABK54280.1); *Capitella *Pax3/7 (ABC68267.1); *Ciona *Otx (NP_001027662.2); *Drosophila *aristaless (NP_722629.1); *Drosophila *orthodenticle (CAA41732.1); *Drosophila *orthopedia (NP_001097388.2); *Drosophila *paired (NP_523556.1); *Euperipatoides *otd (ABY60730.1); *Hydroides *Otx (ABK76302.1); *Mus *ARX (NP_031518.2); *Mus *orthopedia (NP_035151.1); *Mus *Otx1 (NP_035153.1); *Mus *Otx2 (NP_659090.1); *Mus *Pax3 (NP_032807.3); *Mus *Pax7 (NP_035169.1); *Octopus *orthodenticle-like (AAZ99218.1); *Patella *orthodenticle (AAM33144.1); *Patella *orthopedia (AAM33145.1); *Platynereis *ARX (ADG26723.1); *Platynereis *orthopedia (ABR68849.1); *Platynereis *Otx (CAC19028.1); *Saccoglossus *orthodenticle (NP_001158360.1); *Strongylocentrotus *Otx (NP_999753.2); *Terebratalia *Otx (HQ679622)

### Photoresponse behavior assay

Middle gastrula stage embryos were placed in a phototaxis chamber with a 1.7 cm diameter. The chamber was mounted on a stereo microscope with a red filter over the transmitted light base. The chamber was agitated prior to the initiation of transmitted illumination to evenly distribute the embryos. Embryos were exposed to transmitted red-light illumination for five minutes prior to initiation of directional illumination. Embryos were then exposed to lateral directed illumination from a cold white-light source for 30 minutes, followed by an additional 5 minutes with only transmitted red-light illumination. Time-lapse images were required during the entire experiment at a rate of two frames per second. Distribution and movement of embryos was measured with ImageJ [52] and Volocity (PerkinElmer, Waltham, MA, USA) software. Embryos within 1 mm of the sides of the chamber were excluded measurements to avoid possible edge effects.

## Abbreviations

BCIP: 5-Bromo-4-chloro-3-indolyl phosphate; cPRC: ciliary photoreceptor cell; DIC: differential interference contrast; HNPP: 2-hydroxy-3-naphtoic acid-2'-phenylanilide phosphate; NBT: Nitroblue tetrazolium chloride; rPCR: rhabdomeric photoreceptor cell; TEM: transmission electron microscopy;

## Competing interests

The authors declare that they have no competing interests.

## Authors' contributions

YJP participated in the design of the study, performed molecular cloning, sequence analyses, gene expression protocols, microscopic imaging and photoresponse behavior analyses, and drafted the manuscript. NF performed electron microscopy. AH participated in the design of the study, performed molecular cloning, sequence analyses, gene expression protocols and microscopic imaging, and assisted in drafting the manuscript. MQM participated in the design of the study and assisted in drafting the manuscript. CL participated in the design of the study, performed electron microscopy, and assisted in drafting the manuscript.

## Supplementary Material

Additional file 1 **Presumed light-perceptive cilium of the pigment cell in a larval eye of *Terebratalia***. **(A-I) **Series of aligned sections to illustrate the ciliary membrane forming the stack of membranes (m) in the optical cavity enclosed by the lens (ls) and the pigment granules (pg). **(J) **Close-up of the membrane stack (m) showing the invagination of the ciliary membrane to enlarge its surface (arrow). **(K) **Cross-section of the same cilium showing its 9 × 2 + 2 microtubule pattern. Scale bars: 0.5 μm. Click here for file

Additional file 2 **Alignment of deduced amino acid sequences for C-terminus of *Terebratalia *c-opsin and representative c-opsins from other taxa**. Alignment of the of *Terebratalia *c-opsin C-terminus to the C-termini of other c-opsins. The conserved C-terminus domain is required for localization of c-opsin proteins to the ciliary compartment, through binding to the light chain dynein Tctex-1 [60]. Click here for file

Additional file 3 **Middle gastrula swimming prior to directional illumination**. Time lapse imaging of middle gastrula stage embryos swimming in the phototaxis chamber prior to the initiation of directional illumination. Embryos are evenly distributed throughout the chamber. Frame rate is 5× faster than real-time. Click here for file

Additional file 4 **Middle gastrula swimming with directional illumination**. Time lapse imaging of middle gastrula stage embryos swimming in the phototaxis chamber 20 minutes after the initiation of directional illumination. Embryos are clustered on the left side of the chamber, closest to the source of directional illumination. Frame rate is 5× faster than real-time. Click here for file

Additional file 5 **Middle gastrula swimming after directional illumination**. Time lapse imaging of middle gastrula stage embryos swimming in the phototaxis chamber 10 minutes after the cessation of directional illumination. Embryos have returned to an even distribution throughout the chamber. Frame rate is 5× faster than real-time. Click here for file
