# Supplementary figures and images for: Ciliary photoreceptors in the cerebral eyes of a protostome larva
Source: EvoDevo. 2011 Mar 1;2:6. doi: 10.1186/2041-9139-2-6 (PMC3062599; doi:10.1186/2041-9139-2-6)

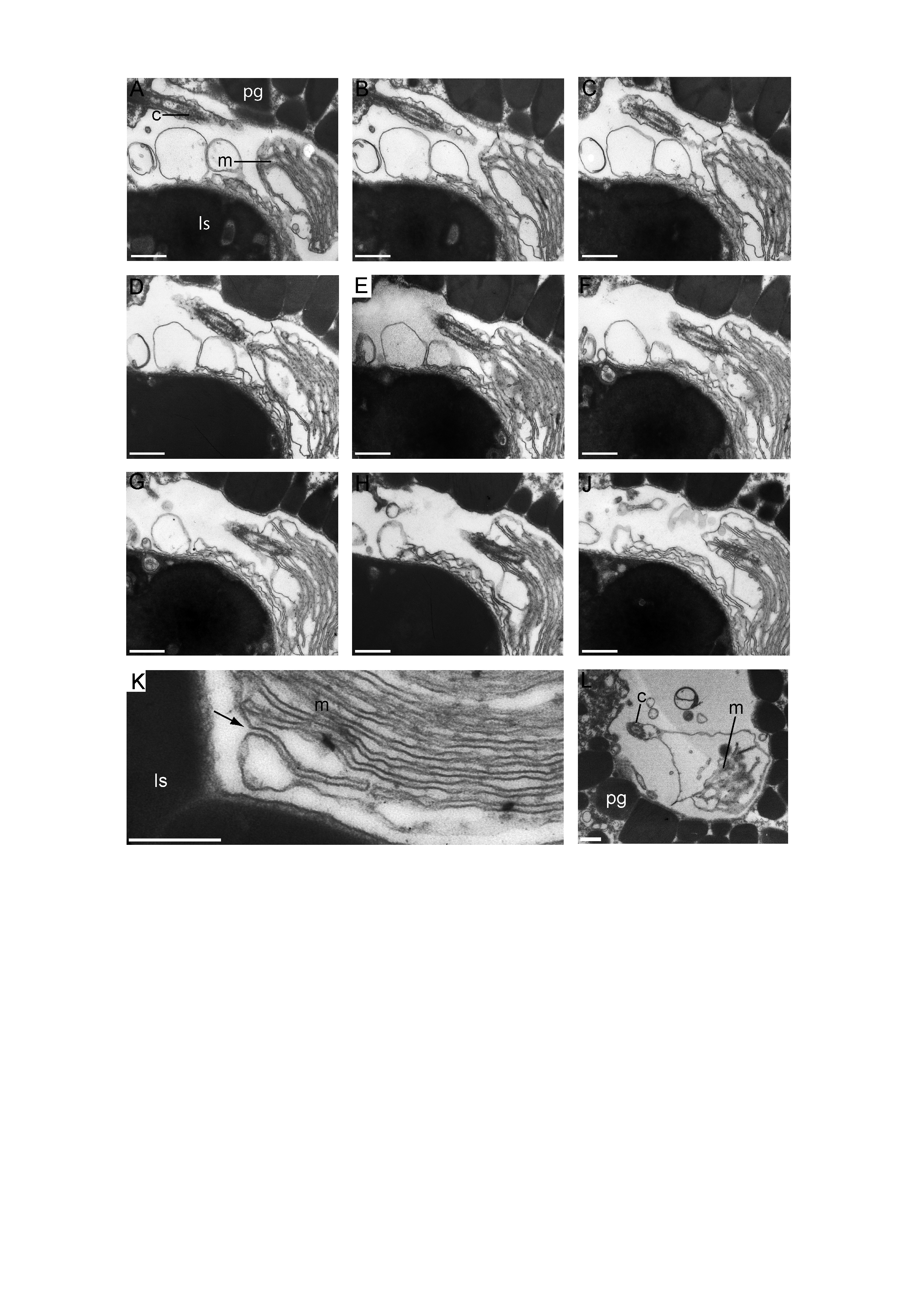

Supplement: Additional file 1 — Presumed light-perceptive cilium of the pigment cell in a larval eye of Terebratalia. (A-I) Series of aligned sections to illustrate the ciliary membrane forming the stack of membranes (m) in the optical cavity enclosed by the lens (ls) and the pigment granules (pg). (J) Close-up of the membrane stack (m) showing the invagination of the ciliary membrane to enlarge its surface (arrow). (K) Cross-section of the same cilium showing its 9 × 2 + 2 microtubule pattern. Scale bars: 0.5 μm. [file 2041-9139-2-6-S1.tiff]
