# Supplementary material for: Ciliary photoreceptors in the cerebral eyes of a protostome larva
Source: EvoDevo. 2011 Mar 1;2:6. doi: 10.1186/2041-9139-2-6 (PMC3062599; doi:10.1186/2041-9139-2-6)
Supplement: Additional file 2 — Alignment of deduced amino acid sequences for C-terminus of Terebratalia c-opsin and representative c-opsins from other taxa. Alignment of the of Terebratalia c-opsin C-terminus to the C-termini of other c-opsins. The conserved C-terminus domain is required for localization of c-opsin proteins to the ciliary compartment, through binding to the light chain dynein Tctex-1 [60]. [file 2041-9139-2-6-S2.PDF]

***Terebratalia* c-opsin**	N	A	V	E	V	*
Platynereis c-opsin	Q	V	A	A	T	*
Homo encephalopsin	Q	V	R	P	L	*
Ciona opsin1	K	V	A	P	A	*
Geotria LWS	S	V	S	P	A	*
Latimeria Rh1	Q	V	S	P	A	*
Xenopus green-rod opsin	Q	I	A	P	S	*
